# Soluble Quartz

**Published:** 1859-06

**Authors:** Geo. Watt


### DENTAL REGISTER OF THE WEST.

VOL. XII.—JUNE, 1859.—NO. 4.

### Original Essays and Communications.

### "SOLUBLE QUARTZ."

BY GEO. WATT.

THE readers of the "*Register*" have all *heard* of "soluble quartz." It is proposed now to inquire if any of them have *seen* it; and, if they have, what of it? What is to be done with it? who is going to do it? and when will he get it done? A long time ago one of our teachers told us, to prove how soon teeth would be filled with "silicious compounds," that he had a gallon of silex, or quartz, in a soft state, "like jelly," and that as soon as he could devise a method of "hardening" it again, he had no doubt that the desire of all dentists would be reached. Various hints have since dropped from the tongues of hopeful aspirants, all pointing to the *silicious* desideratum; and, finally, we hear of "dissolving quartz," in company with an explosive warning not "to steal one's thunder."

Well, quartz is crystalized silica, or silicic acid. As a mineral, it is well known. It is composed of oxygen and silicon, and is as really an acid as the well known sulphuric acid. The reason it does not taste sour, and otherwise manifest the ordinary properties of acids, is that it is insoluble.

Like all acids, it has an affinity for, and unites with alkaline bases, such as potash, soda, and the like. On account of its solidity and insolubility, heat is necessary to its speedy combination with an alkali. The compounds formed by its union with bases are called *silicates*. The different varieties of glass are all silicates.

The alkaline bases are usually *protoxyds*, that is, they are mostly composed of one equivalent of oxygen united with one equivalent of some other element. Thus, potash is composed of one equivalent of potassium and one of oxygen; and it may serve as a type of the basic oxyds. Now, the acids prefer the alkalies to other oxyds, as a general rule; but, when deprived of these they will unite with the others. Water is the *protoxyd of hydrogen*, and we often find the strong acids combined with it. Liquid sulphuric acid is one equivalent of the *pure* acid, combined with one of water. The same is true of nitric acid. When the latter acid unites with potash, the compound is called *nitrate of potash;* and, on the same principle, when it combines with water, the compound should be called *nitrate of water*, instead of "liquid nitric acid."

Bearing these facts in mind, by way of illustration, it will be easier to understand just what is meant by soluble, or "dissolving quartz." If powdered quartz, or silicic acid, be mixed with soda or potash, or with their carbonates, and heated to a red heat, the two substances combine. If a carbonate be used, carbonic acid escapes as a gas. The properties of the compounds thus formed vary with the proportions of the materials used. If one part of the powdered quartz be mixed with three of carbonate of potash, and the mixture heated to redness in a crucible, a soluble glass, or silicate of potash, is formed, the silicic acid being able, by the aid of heat, to take the potash from the carbonic acid.

The glass thus formed is easily pulverized, and is readily dissolved in water. When dissolved, if a strong acid, as the nitric or sulphuric, is added to the solution, a gelatinous precipitate is formed. This is the "gelatinous silex," or "soluble quartz." If it be heated to redness, pure silicic acid, in the form of a fine powder, is obtained. This gelatinous precipitate may be obtained by a variety of processes; but the one here noticed is as convenient as any other.

But what is this gelatinous precipitate? Is it silicic acid or quartz, dissolved, or partially dissolved, in water? In answer, let us endeavor to comprehend the chemical changes which take place in the process of its formation.

The soluble glass is *silicate of potash;* and a so-called concentrated acid, which may be added to the solution of the glass to cause the precipitation, is *nitrate of water.* In the change which is effected, we have a case of what is called double elective affinity. The reäctions will be better understood by a diagram:

| | | | |
|---|---|---|---|
| Silicate of potash, | Silicic acid,——— | | Silicate of water. |
| | Potash, | Nitrate of potash. | |
| Nitrate of water, | Nitric acid, | | |
| | Water,——— | | |

Those familiar with chemical notation will understand the matter as well, or better, from an equation:

$$KO,SiO_3+HO,NO_5=HO,SiO_3+KO,NO_5$$

From this it appears that the quartz, instead of being dissolved in water, is chemically combined with it; and this compound retains its gelatinous form with a goodly degree of tenacity. Its plasticity would enable us to insert it in a cavity in a tooth; but in its gelatinous state it would avail nothing. And if we could devise a ready method of *removing* the water from it, thus *decomposing* the silicate, powdered quartz would be our sole reward.

Soluble *silicates* are common; but soluble *quartz* is, probably, somewhat scarce; and, were it otherwise, it would probably dissolve in the fluids of the mouth as readily as in water.

It is probable that teeth will be filled with gold for a while yet; but we are waiting patiently "to see what we shall see."

